# Genetic diversity of Brazilian *Bacillus thuringiensis* isolates with toxicity against *Aedes aegypti* (Diptera: Culicidae)

**DOI:** 10.1038/s41598-022-18559-0

**Published:** 2022-08-24

**Authors:** Geysla da Costa Fernandes, Dalton Kaynnan de Prado Costa, Nayanne Santos de Oliveira, Emanuelle Cristine Pereira de Sousa, Déborah Heloísa Bittencourt Machado, Ricardo Antonio Polanczyk, Herbert Álvaro Abreu de Siqueira, Maria Cleoneide da Silva

**Affiliations:** 1 PostGraduate Program in Biodiversity, Environment and Health, Center for Higher Studies of Caxias (CESC), State University of Maranhão (UEMA), Praça Duque de Caxias, Morro do Alecrim, S/N, Caxias, MA 65604-380 Brazil; 2 Laboratory of Entomopathogenic Bacteria and Molecular Markers, Center for Higher Studies of Caxias (CESC), State University of Maranhão (UEMA), Praça Duque de Caxias, Morro do Alecrim, S/N, Caxias, MA 65604-380 Brazil; 3 Postgraduate Program in Animal Science, State University of Maranhão (UEMA), University City Paul VI, Avenida Lourenço Vieira da Silva 1000, Jardim São Cristóvão, São Luís, MA 65055-310 Brazil; 4 Postgraduate Program in Agronomy (Agricultural Entomology), São Paulo State University (UNESP), School of Agricultural and Veterinarian Sciences, Jaboticabal, 14884‑900 Brazil; 5 Department of Agricultural Production Sciences, São Paulo State University (UNESP), School of Agricultural and Veterinarian Sciences, Jaboticabal, 14884‑900 Brazil; 6 Department of Agronomy (Entomology), Federal Rural University of Pernambuco (UFRPE), Recife, PE 52171-900 Brazil; 7 Department of Chemistry and Biology, Laboratory of Entomopathogenic Bacteria and Molecular Markers, Center for Higher Studies of Caxias, State University of Maranhão, Praça Duque de Caxias, Morro do Alecrim, S/N, Caxias, MA 65604-380 Brazil

**Keywords:** Biological techniques, Genetics, Molecular biology

## Abstract

*Bacillus thuringiensis* (Bt) isolates native to Maranhão (BtMA) that are highly toxic to *Aedes aegypti* larvae and seven standard subspecies of Bt were analyzed for genetic diversity using the rep-PRC technique with BOX, ERIC, REP, MB1, and GTG_5_ markers. The rep-PCR technique is considered an extremely reliable, reproducible, fast and highly discriminatory technique that may be used even among populations of the same species. These five markers revealed a total of 38 polymorphic DNA fragments for 30 BtMA isolates. Eight groups were obtained with the dendrogram generated through Pearson's correlation analysis, with four groups formed only with BtMA isolates and four comprised of isolates of BtMA and the standard subspecies toxic to dipterans and lepidopterans. Despite the high genetic diversity of BtMA, a low correlation between the collection site, gene content and mortality against *A. aegypti* larvae was evidenced. The clustering of the standard subspecies of Bt that were toxic against dipterans with BtMA isolates confirm the mosquitocidal action of the native isolates from Maranhão, and they can be used as an alternative for *A. aegypti* control and other insects of medical importance and for the control of agricultural pests.

## Introduction

*Bacillus thuringiensis* (Bt) is a gram-positive, aerobic, rod-shaped and spore-forming entomopathogenic bacterium that is highly effective in controlling immature forms of mosquitoes, since several strains produce protein crystals containing δ-endotoxin (Cry and Cyt) with mosquitocidal action^1–5^.

Several subspecies of Bt have been reported to be highly toxic against dipteran insects, such as *Bacillus thuringiensis israelensis* (Bti), *Bacillus thuringiensis entomocidus* (Bte), *Bacillus thuringiensis sotto* (Bts), *Bacillus thuringiensis jegathesan* (Btj), *Bacillus thuringiensis darmstadiensis* (Btd), *Bacillus thuringiensis medellin* (Btm), *Bacillus thuringiensis fukuokaensis* (Btf), and *Bacillus thuringiensis higo* (Bth)^6–13^. *Bacillus thuringiensis israelensis* is considered the most toxic to dipteran larvae^1,3,14^. The mosquitocidal activity of Bti is due to pesticidal proteins encoded by the *cry4Aa*, *cry4Ba*, *cry11Aa*, *cyt1Aa*, *cry10Aa* and *cyt2Ba* genes^1,5,15^.

Molecular typing has been used to characterize and discriminate species belonging to *Bacillus*, in addition to monitoring the evolution and phylogeny of the group^16–19^. Thus, the rep-PCR technique is widely used to investigate specific genetic relationships between Bt isolates^20–25^.

The rep-PCR technique uses primers specific to the *Bacillus cereus* group and is considered an extremely reliable, reproducible, fast and highly discriminatory technique that may be used even among populations of the same species^26–30^ or even among microorganisms^20,23,27,30^.

The objective of the current study was to estimate the genetic diversity of Bt isolates from the Entomopathogenic Bacteria Collection of Maranhão (CBENMA) and the Entomopathogenic Bacilli Bank of Maranhão (BBENMA) that are toxic to *A. aegypti* larvae^31–33^ using the repetitive sequences BOX, ERIC, REP, MB1, and GTG_5_, to evaluate the association between Bt isolate clusters and standard subspecies of this bacterium, geographic regions of collection, mortality against *A. aegypti* larvae, and amplification of dipteran-specific genes (*cry* and *cyt* genes).

## Results

**Figure 1:**
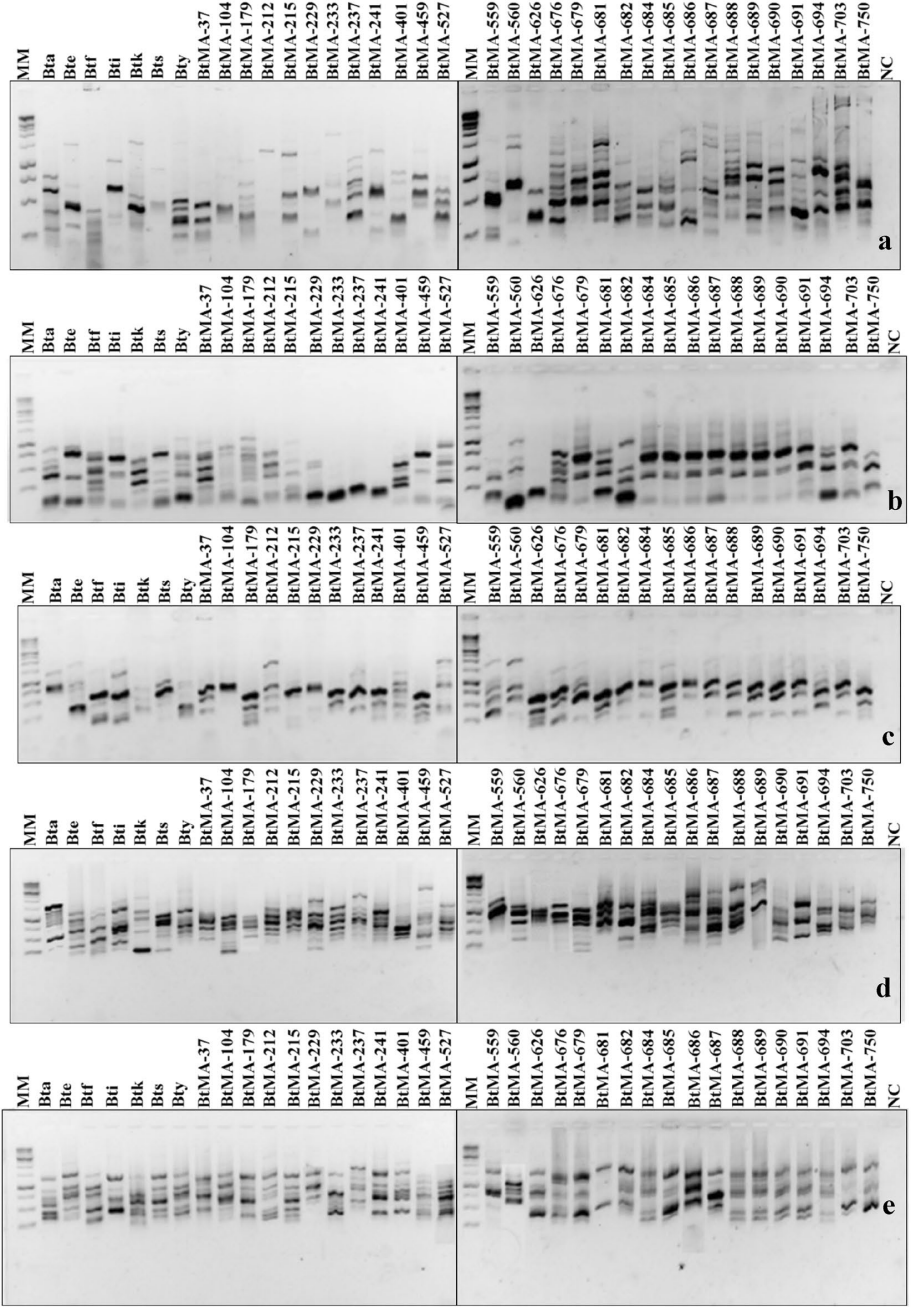
Rep-PCR fingerprint showing the amplification of the BOX (**a**), ERIC (**b**), REP (**c**), MB1 (**d**) and GTG_5_ (**e**) molecular markers in 30 isolates of *Bacillus thuringiensis* and seven standard subspecies. MM: Molecular marker; Bta: *B. thuringiensis aizawai*; Bte: *B. thuringiensis entomocidus*; Btf: *B. thuringiensis fukuokaensis*; Bti: *B. thuringiensis israelensis*; Btk: *B. thuringiensis kurstaki*; Bts: *B. thuringiensis sotto*; Bty: *B. thuringiensis yunnanensis*; BtMA: *Bacillus thuringiensis* from Maranhão; NC: negative control.

The diversity of *Bacillus thuringiensis* isolates native to Maranhão (BtMA) was determined by rep-PCR, and a complex fingerprint pattern was obtained for the 30 BtMA isolates with the BOX, ERIC, REP, MB1 and GTG_5_ primers. The results revealed a total of 38 polymorphic DNA fragments (Fig. 1).

The number of fragments per isolate for the BOX primer ranged from one to seven, with the smallest fragment of 100 bp and the largest exceeding 10.000 bp, with a total of 24 electrophoretic patterns (Fig. 1a). The ERIC and REP primers provided fragments ranging from one to six per isolate. For the ERIC primer, the base pairs ranged from 250 to 1250 bp, and for REP, they ranged from 250 to 2000 bp, with a total of 13 and 12 electrophoretic patterns, respectively (Fig. 1b,c). The MB1 primer provided between one to eight fragments and a total of 31 electrophoretic patterns with fragment sizes between 250 and 6000 bp (Fig. 1d). Regarding the GTG_5_ primer, the BtMA isolates provided fragments of repetitive sequences that ranged from 2 to 11 bands, with the majority being similar, from 200 to 3000 bp, with a total of 11 electrophoretic patterns (Fig. 1e). Although some isolates shared the same bands, most of them presented different fragment profiles.

**Figure 2:**
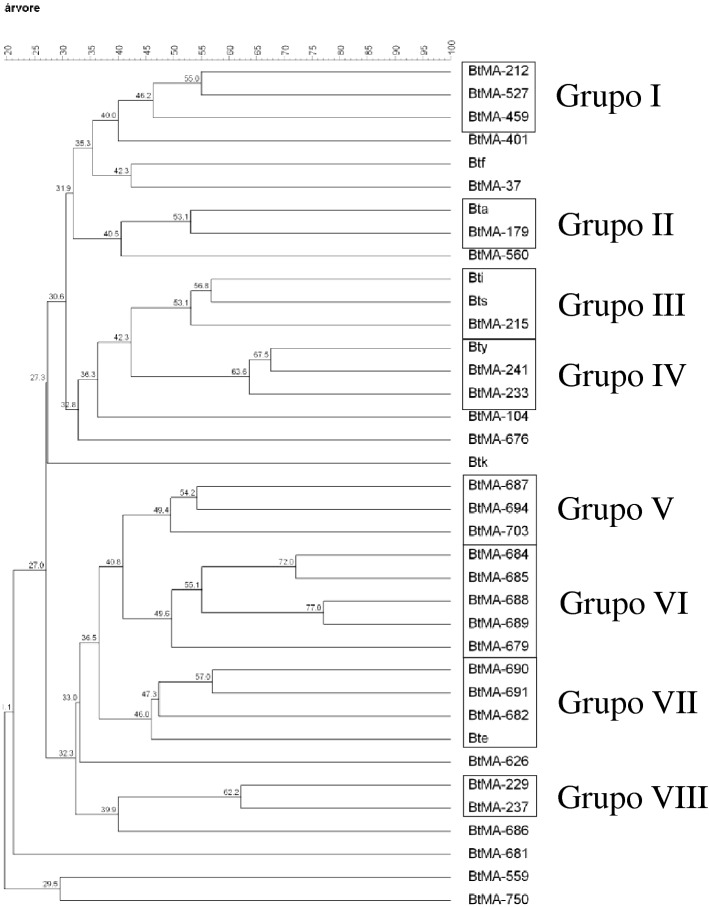
Dendrogram produced by cluster analysis (UPGMA) based on the Jaccard coefficient by using the BOX, ERIC, REP, MB1 and GTG_5_ molecular markers and molecular fingerprint of 30 isolates of *Bacillus thuringiensis* from diverse locations in Maranhão and seven standard subspecies of *B*. *thuringiensis*.

A dendrogram was generated through Pearson's correlation for genetic diversity analyses using the sequences BOX, ERIC, REP, MB1, and GTG5, with an average distance of 45% (cut-off point for the formation of groups with less genetic variation), in which eight groups (designated I, II, III, IV, V, VI, VII and VIII) were obtained, which comprised 67.5% of the isolates (Fig. 2). The other Bt isolates showed an average distance greater than the cut-off point.

Groups I, V, VI and VIII were formed only with BtMA isolates (Fig. 2). Group I (BtMA-212, BtMA-527 and BtMA-459) and group V (BtMA-687, BtMA-694 and BtMA-703) included isolates obtained from different biomes. The BtMA-212 isolate obtained from Amazonia grouped with the BtMA-527 and BtMA-459 isolates from the Cerrado. These three isolates, in addition to presenting 100% mortality to *A. aegypti* larvae in the second evaluation (48 h), amplified the *cry* and *cyt* genes.

Groups VI and VIII were formed by isolates from the same biome, with isolates of group VI (BtMA-679, BtMA-684, BtMA-685, BtMA-688 and BtMA-689) all from the Cerrado and those from group VIII (BtMA-229 and BtMA-237) from the Amazon. All isolates in group VI caused 100% mortality within a 24-h period and amplified all analyzed dipteran-specific genes, with the exception of the BTMA-689 isolate, which did not present the *cry11Ba* gene. For the genetic profile of isolates from group VIII, it was found that they did not share any of the *cry* and *cyt* genes analyzed. An *A. aegypti* larval mortality of 100% was caused by isolates of BtMA in this group at 48 h.

Groups II, III, IV and VII comprised isolates of BtMA and the standard subspecies. The BtMA-179 isolate that grouped with the subspecies *Bacillus thuringiensis aizawai* (Bta) (group II) was isolated from the soil of the Amazon biome, caused 100% mortality in 48 h and amplified only the dipteran-specific *cry4Ba* gene, and the BtMA-233 and BtMA-241 isolates grouped with subspecies *Bacillus thuringiensis yunnanensis* (Bty) (IV) were isolated from the soil of the Amazon and Cerrado biomes, respectively. These three isolates were toxic to *A. aegypti* larvae over a period of 48 h, and isolates BtMA-233 and BtMA-241 presented the *cyt2Aa* gene in common. The subspecies Bta and Bty are toxic to insects of the order Lepidoptera^34–36^, which suggests that isolates from groups II and IV may also be active against insects of that order.

The BtMA-215 isolate, obtained from Amazonian soil, did not amplify any of the analyzed genes, caused 100% mortality in *A. aegypti* larvae in 48 h, and grouped with two subspecies, *Bacillus thuringiensis. israelensis* (Bti) and *Bacillus thuringiensis sotto* (Bts) (group III). These subspecies possibly confirm the mosquitocidal action of the isolate BtMA-215. However, more research should be carried out with the isolate BtMA-215 to predict it as a strain with potential for the control of dipterans and lepidopterans.

The isolates BtMA-682, BtMA-690 and BtMA-691 were found in group VII, all from the Cerrado soil, with different mortality rates and genetic profiles, and grouped with the subspecies *Bacillus thuringiensis entomocidus* (Bte), which is at the same time effective against insects of the orders Diptera and Lepidoptera^8,37^. It is worth mentioning that the BtMA-690 isolate amplified all the *cry* and *cyt* genes analyzed and provided 100% mortality of *A. aegypti* larvae in 24 h.

## Discussion

The repetitive element palindromic-polymerase chain reaction (rep-PCR) technique has been used to investigate the interspecific and intraspecific genetic diversity of Bt isolates, allowing us to correlate them with the geographic regions of collection, the origin of the substrate, genetic characteristics, and rates of mortality and toxicity against insect^22,23,29,38,39^.

In this research, the genetic diversity of BtMA isolates, collected in different biomes, with a high mortality against larvae of *A. aegypti* and that amplified for *cry* and *cyt* dipteran-specific genes^31–33^, was studied using group analysis with data obtained with the BOX, ERIC, REP, MB1 and GTG_5_ primers.

The BOX and MB1-PCRs were the most informative, producing the most complex fragment patterns^20,25,40^. Robust and, commonly, highly complex fingerprints are obtained using the BOX primer^41,42^. The MB1 primer generated extremely complex fingerprints, showing that almost all 30 BtMA isolates had their own unique MB1-PCR standard, suggesting high genetic variability among the isolates^21,40^.

*Bacillus thuringiensis* is a genetically diverse species, so its large number of strains can form several different profiles according to their genetic profile^43^. The high genetic variability of Bt may be related to both the influence of geographic and ecological factors^22–24^ because this bacterium is isolated from several substrates and has a wide range of target hosts.

Several studies have reported that ERIC markers are more informative than REP markers in discriminating Bt strains^20,23,39,44^, as was also verified in this research, suggesting that ERIC-like sequences may be more widely distributed than REP-like sequences in Bt^44^.

Among all the markers, GTG_5_ was not as effective in discriminating between isolates of BtMA, indicating low genetic diversity. A similar result was verified when this marker was used to determine the diversity among the different *Bacillus* species^25,45^.

It was possible to verify that groups VI, VII and VIII included isolates of BtMA from the same biome. The similarity of the BtMA isolates from the Cerrado (groups VI and VII) and the Amazon (VIII) biomes may be related to the geographic location. However, groups I, IV and V included isolates of BtMA from the Cerrado with other biomes. The tendency to form groups or subgroups in relation to the similarity of Bt according to geographic origin, using the Rep-PCR technique, is quite controversial.

Huerta et al.^38^, using REP primers, evaluated the genetic diversity of 53 Bt isolates collected in soil from different geographical regions of Peru and correlated the similarity of these isolates according to geographic origin. Similarly, Da Silva and Valicente^20^ pointed out that the similarity of Bt isolates may be related to geographic location when they used the ERIC, REP and BOX primers simultaneously. Katara et al.^44^ observed that the REP-PCR and ERIC-PCR patterns, generated with 113 Bt native isolates from diverse habitats of India, formed four main groups with isolates of diverse origin.

All isolates of BtMA from the VI group (BtMA-684, BtMA-685, BtMA-688, BtMA-689, and BtMA-679) presented the same mortality rate (100% in 24 h), and all isolates of BtMA from the I, II and VIII groups caused 100% mortality within 48 h. Groups V and VII included isolates with different mortality rates (24 and 48 h). However, further studies are needed to confirm the hypothesis that similarity is related with the pathogenicity.

When correlating the mortality of the BtMA isolates and the site of origin, only group VI, constituted by the isolates BtMA-684, BtMA-685, BtMA-688, BtMA-689 and BtMA-679, showed a genetic relationship. This low correlation was also verified by Huerta et al.^38^ when verifying subgroups of Bt formed according to the geographical origin and potential use against *A. aegypti*.

Likewise, Da Silva and Valicente^20^ and Machado et al.^23^, comparing the genetic relationship of Bt isolates toxic to *Spodoptera* spp. and the collection sites of these isolates reported a low correlation. In addition, in agreement with Reyes-Ramirez and Ibarra^43^ and Da Silva and Valicente^20^, the genomic relationship between Bt strains is not defined only by their specific toxicity but by several characteristics, such as the content of the *cry* gene, crystal morphology and plasmid pattern.

The BtMA isolates showed a great diversity of the diptera-specific genes *cry4*, *cry10*, *cry11*, *cyt1* and *cyt2*^31–33^. Groups V and VI included BtMA isolates with all the investigated genes, with the exception of the BtMA-694 isolate from group V, which did not amplify the *cyt2B*a gene, and the BtMA-689 isolate from group VI, which did not amplify the *cry11Ba* gene. The great diversity of genes encoding mosquito-specific toxins in BtMA isolates, presenting the same genetic content as Bti, represents a great opportunity to control vector insects and enables strategies to manage the evolution of insect resistance to Bt pesticidal proteins.

One factor that may or may not explain the correlation with the Bt collection sites is that the diversity may also be associated with the region's climate, soil conditions where the samples were collected, and the influence of other microbial communities present in the region^24^.

Interestingly, the isolates of group V (BtMA-687 BtMA-694 BtMA-703) did not show any relation to the geographic origin and mortality of the *A. aegypti* larvae, and the isolates of VI (BtMA-684, BtMA-685, BtMA-688, BtMA-689 and BtMA-679) were all from the same biome and caused 100% mortality to *A. aegypti* larvae in the first evaluation (24 h). Based on these results, the correlation between the genetic relationship of BtMA isolates and their investigated gene content is very low. Lima et al.^39^ highlighted that repetitive sequences showed no relation to the types of pesticidal protein produced by Bt, which demonstrates that the majority of clusters that occur between isolates that do not carry the same combinations of dipteran-specific genes.

For the groups formed with the standard subspecies and the BtMA isolates (groups II, III, IV and VII), it was possible, in addition to confirming the toxic activity of these isolates against *A. aegypti* larvae, to suggest the potential of the BtMA isolates for the control of insects of the order Lepidoptera. However, it will be necessary to carry out bioassays with larvae of pest lepidopterans and to characterize these isolates with lepidopteran-specific genes of Bt.

An interesting fact was the grouping of the isolate BtMA-215 with the subspecies Bti (group III). Unlike the subspecies Bti, this isolate caused mortality to *A. aegypti* larvae within 48 h and did not amplify any of the diptera-specific *cry* and *cyt* genes selected for this study. However, Soares-da-Silva et al.^32^ reported that the BtMA-215 isolate amplified the *cry32* gene. There are 29 Cry32 proteins^46^, and according to Van Frankenhuyzen^47^ and Rajchanuwong et al. ^48^, these proteins are toxic to insects of the order Diptera, especially mosquito larvae.

In this work, we provided evidence of high genetic variability of Bt isolates native to Maranhão (BtMA) and toxic to *A. aegypti*, providing important information about their phylogenetic relationships and similarity with the standard subspecies (Bti, Bta, Bte, Bty, and Bts).

In addition, the clusters with the standard subspecies of Bt toxic to dipterans with BtMA isolates confirm the mosquitocidal action of the native isolates from Maranhão and that they can be used as an alternative for control of *A. aegypti* and other insects of medical importance. This fact also demonstrated that isolates that grouped with subspecies toxic to lepidopterans, such as Bta and Bty, could constitute new tools in integrated pest management, providing different Cry pesticidal proteins than those expressed in ordinary Bt formulations^49^.

We must note that detailed information on the Bt *cry* gene profile is highly important not only for approaches to insect resistance management and host spectrum^47,50,51^ but also for providing important insights into Bt formulation because different pesticidal proteins can require specific fermentation conditions, such as dissolved oxygen and temperature^52,53^.

## Methods

### *Bacillus thuringiensis* isolates

Thirty *Bacillus thuringiensis* isolates native to Maranhão (BtMA) obtained from soil samples, with high toxicity to *A. aegypti* larvae and that amplified for *cry* (*cry4Aa*, *cry4Ba*, *cry10Aa*, *cry11Aa* and *cry11Ba)* and *cyt* (*cyt1Aa, cyt1Ab*, *cyt2Aa* and *cyt2Ba*) genes^31–33^, and seven standard subspecies of Bt were used in this survey (Tables 1 and 2). All these isolates are stored in CBENMA located in the Laboratory of Entomopathogenic Bacteria and Molecular Markers (BEMMOL) at the Centro de Estudos Superiores de Caxias of Universidade Estadual do Maranhão (CESC/UEMA).

**Table 1:** Isolates of *Bacillus thuringiensis* of Maranhão, collection biome, molecular characterization of dipteran-specific genes, and mortality rates against *Aedes aegypti* larvae.

N°	Isolates	Biome	Dipteran-specific genes									Pathogenicity
			*cry4Aa*	*cry4Ba*	*cry10Aa*	*cry11Aa*	*cry11Ba*	*cyt1Aa*	*cyt1Ab*	*cyt2Aa*	*cyt2Ba*	
1	BtMA-37^1^	Amazônia	+									100% (24 h)
2	BtMA-104^2^	Cerrado						+			*	100% (24 h)
3	BtMA-179^1^	Amazônia		+								100% (48 h)
4	BtMA-212^3^	Amazônia		+	+	+			+	+		100% (48 h)
5	BtMA-215^1^	Amazônia										100% (48 h)
6	BtMA-229^1^	Amazônia		+						+		100% (48 h)
7	BtMA-233^1^	Amazônia								+	+	100% (48 h)
8	BtMA-237^1^	Amazônia						+	+			100% (48 h)
9	BtMA-241^1^	Cerrado						+		+		100% (48 h)
10	BtMA-401^2^	Cerrado									*	100% (24 h)
11	BtMA-459^1^	Cerrado		+						+		100% (48 h)
12	BtMA-527^1^	Cerrado						+	+			100% (48 h)
13	BtMA-559^1^	Cerrado								+		100% (48 h)
14	BtMA-560^2^	Cerrado				+					*	100% (24 h)
15	BtMA-626^1^	Cerrado							+			100% (48 h)
16	BtMA-676^1^	Cerrado	+	+	+	+	+	+	+	+	+	100% (48 h)
17	BtMA-679^1^	Cerrado	+	+	+	+	+	+	+	+	+	100% (24 h)
18	BtMA-681^1^	Cerrado		+	+	+		+		+	+	100% (24 h)
19	BtMA-682^1^	Cerrado						+	+	+		100% (48 h)
20	BtMA-684^1^	Cerrado	+	+	+	+	+	+	+	+	+	100% (24 h)
21	BtMA-685^1^	Cerrado	+	+	+	+	+	+	+	+	+	100% (24 h)
22	BtMA-686^1^	Cerrado								+		100% (48 h)
23	BtMA-687^1^	Cerrado	+	+	+	+	+	+	+	+	+	100% (24 h)
24	BtMA-688^1^	Cerrado	+	+	+	+	+	+	+	+	+	100% (24 h)
25	BtMA-689^1^	Cerrado	+	+	+	+		+	+	+	+	100% (24 h)
26	BtMA-690^1^	Cerrado	+	+	+	+	+	+	+	+	+	100% (24 h)
27	BtMA-691^1^	Cerrado		+	+		+		+	+	+	100% (24 h)
28	BtMA-694^1^	Cerrado	+	+	+	+	+	+	+	+		100% (48 h)
29	BtMA-703^1^	Caatinga	+	+	+	+	+	+	+	+	+	100% (24 h)
30	BtMA-750^4^	Amazônia	+	+	+	+	+	+	+	+	*	100% (24 h)

^1^Soares-da-Silva et al.^32^.

^2^Lobo et al.^31^.

^3^BEMMOL and BBENMA collection.

^4^Vieira-Neta et al.^33^.

*Untested gene.

**Table 2:** Subspecies of *Bacillus thuringiensis* used as a standard and their respective insect orders against which they show specific toxicity.

Subspecies	Specificity	References
*B. thuringiensis israelensis* (Bti)^1^	Diptera	Thorne et al.^54^, Bourgouin et al.^6,55^
*B. thuringiensis kurstaki* (Btk)^1^	Lepidoptera	Dankocsik et al.^56^, Moar et al.^57^
*B. thuringiensis aizawai* (Bta)^1^	Lepidoptera	Dequech et al.^35^, Lima et al.^36^
*B. thuringiensis entomocidus* (Bte)^2^	Diptera and Lepidoptera	Shin et al.^37^, Ito et al.^8^
*B. thuringiensis fukuokaensis* (Btf)^2^	Diptera	Ishii and Ohba^58^, Lee and Gill^9^
*B. thuringiensis yunnanensis* (Bty)^2^	Lepidoptera	Balasubramanian et al.^34^
*B. thuringiensis sotto* (Bts)^2^	Diptera and Lepidoptera	Ohgushi et al.^11^, Magda and Moharam^59^

^1^Commercial strains: *B*. *thuringiensis israelensis* sorotipo H-14 (VectorBac®WG), *B*. *thuringiensis kurstaki* (Dipel® WP), *B*. *thuringiensis aizawai* (Xentari®WDG).

^2^Strains kindly provided by the Laboratory of Genetics of Bacteria and Applied Biotechnology of the Faculty of Agricultural and Veterinary Sciences of Jaboticabal, Department of Biology Applied to Agriculture. State Paulista Júlio de Mesquita Filho University. Fonte: LABEM and BEMMOL.

### DNA extraction and PCR conditions

Genomic DNA from BtMA isolates and standard subspecies of Bt was extracted using the Instagene matrix (Bio-Rad) according to the manufacturer's recommendations. PCR was performed in final volume of 20 μL. Each reaction mixture contained 50 ng of genomic DNA of Bt isolates, 1 × buffer, 2 mM MgCl_2_, each dNTP at a final concentration of 200 µM, 1 U of Taq DNA Polymerase and 1 μM each primer (BOX, ERIC, REP, MB1, and GTG_5_) (Table 3). Amplification was accomplished with the Gencycler-G96G thermocycler (Biosystems), following the standard program at 94 °C for 5 min; 36 cycles at 94 °C for 1 min; annealing (with temperatures varying for each primer) for 1 min; extension at 72 °C for 2 min; and final extension at 72 °C for 7 min. The PCR products were visualized in a 1,5% agarose gel using a photo documentation system (L-PIX EX Loccus).

**Table 3:** Sequence of primers for Rep-PCR and respective annealing temperatures.

Sequence (5′–3′)	Annealing temperature (°C)	References
BOXA1R—CTACGGCAAGGCGACGCTGACG	52	Koeuth et al.^60^
ERIC 1R—ATGTAAGCTCCTGGGGATTCAC	52	Versalovic et al.^61^
ERIC 2—AAGTAAGTGACTGGGGTGAGCG	52	
REP1R-I—IIIICGICGICATCIGGC	40	
REP2 I—ICGICTTATCIGGCCTAC	40	
MB1—TGTACATAAGACGAAGCCC	52	Brumlik et al.^40^
GTG5—GTGGTGGTGGTGGTG	40	Versalovic et al.^30^

### Data analysis

The Rep-PCR profiles (banding patterns) obtained with the 30 BtMA isolates and the seven standard subspecies of Bt generated binary matrices that were used as input data into Bionumerics software (Applied Maths, Belgium) after Pearson’s correlation analysis. The Dice similarity coefficient was used to calculate the similarity matrix from binary data. Clustering analysis was performed using this coefficient and UPGMA (unweighted pair-group method with arithmetic mean) with 1000 bootstrapping replicates to evaluate the consistency of the group, and Bionumerics was used to produce both the similarity matrix and dendrogram (Supplementary Fig. S1).

## Supplementary Information

Supplementary Figure S1.
