# Supplementary material for: Genetic diversity of Brazilian Bacillus thuringiensis isolates with toxicity against Aedes aegypti (Diptera: Culicidae)
Source: Sci Rep. 2022 Aug 24;12:14408. doi: 10.1038/s41598-022-18559-0 (PMC9402949; doi:10.1038/s41598-022-18559-0)
Supplement: Supplementary file 1 — Supplementary Figure S1. [file 41598_2022_18559_MOESM1_ESM.pdf]

Figure 1 Rep-PCR fingerprint showing the amplification of the BOX (a), ERIC (b), REP (c), MB1 (d) and GTG_5 (e) molecular markers in 30 isolates of *Bacillus thuringiensis* and seven standard subspecies. MM: Molecular marker; Bta: *B. thuringiensis aizawai*; Bte: *B. thuringiensis entomocidus*; Btf: *B. thuringiensis fukuokaensis*; Bti: *B. thuringiensis israelensis*; Btk: *B. thuringiensis kurstaki*; Bts: *B. thuringiensis sotto*; Bty: *B. thuringiensis yunnanensis*; BtMA: *Bacillus thuringiensis* from Maranhão; NC: negative control.

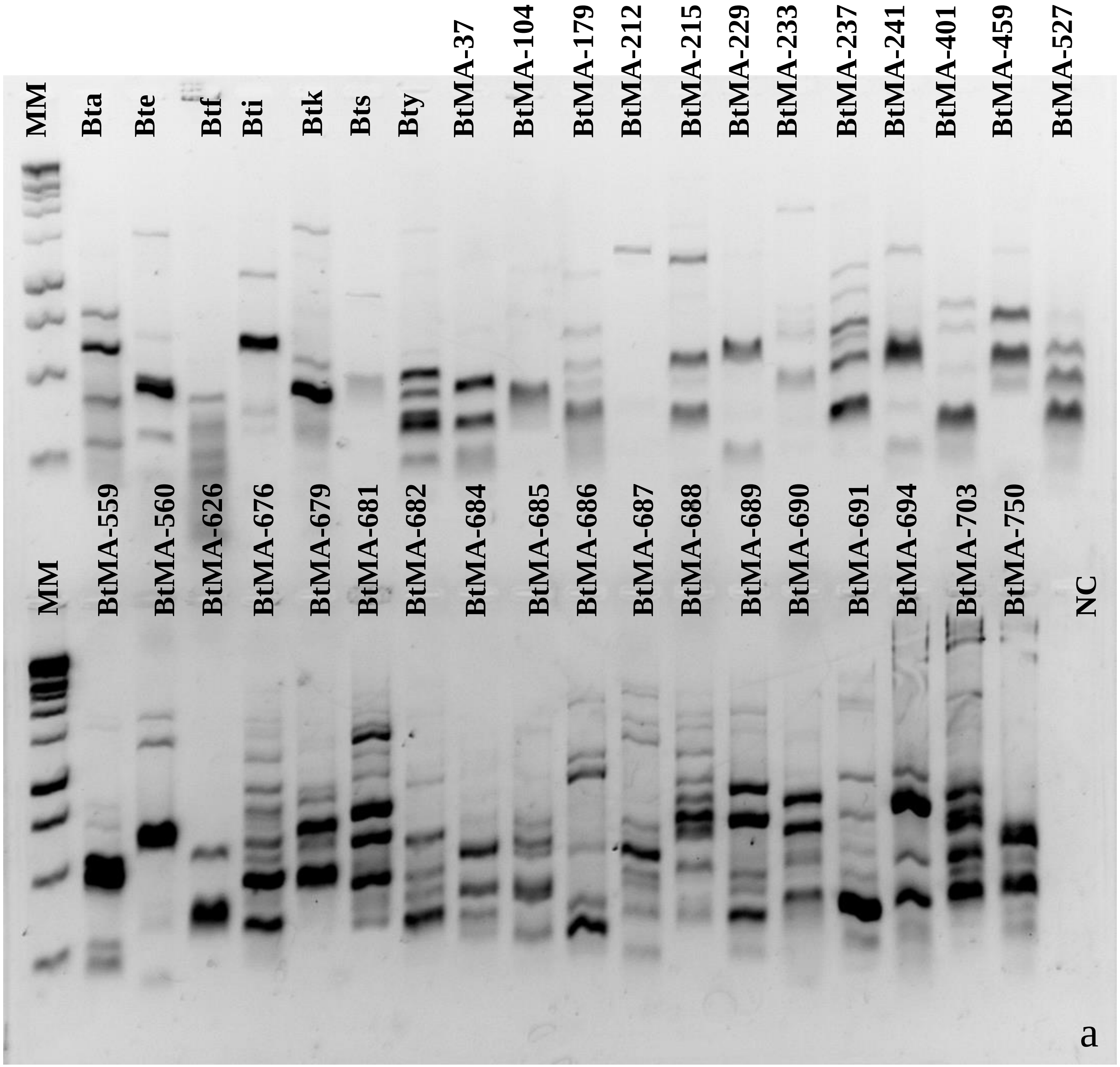
MM
Bta
Bte
Btf
Bti
Btk
Bts
Bty
BtMA-37
BtMA-104
BtMA-179
BtMA-212
BtMA-215
BtMA-229
BtMA-233
BtMA-237
BtMA-241
BtMA-401
BtMA-459
BtMA-527
MM
BtMA-559
BtMA-560
BtMA-626
BtMA-676
BtMA-679
BtMA-681
BtMA-682
BtMA-684
BtMA-685
BtMA-686
BtMA-687
BtMA-688
BtMA-689
BtMA-690
BtMA-691
BtMA-694
BtMA-703
BtMA-750
NC
a

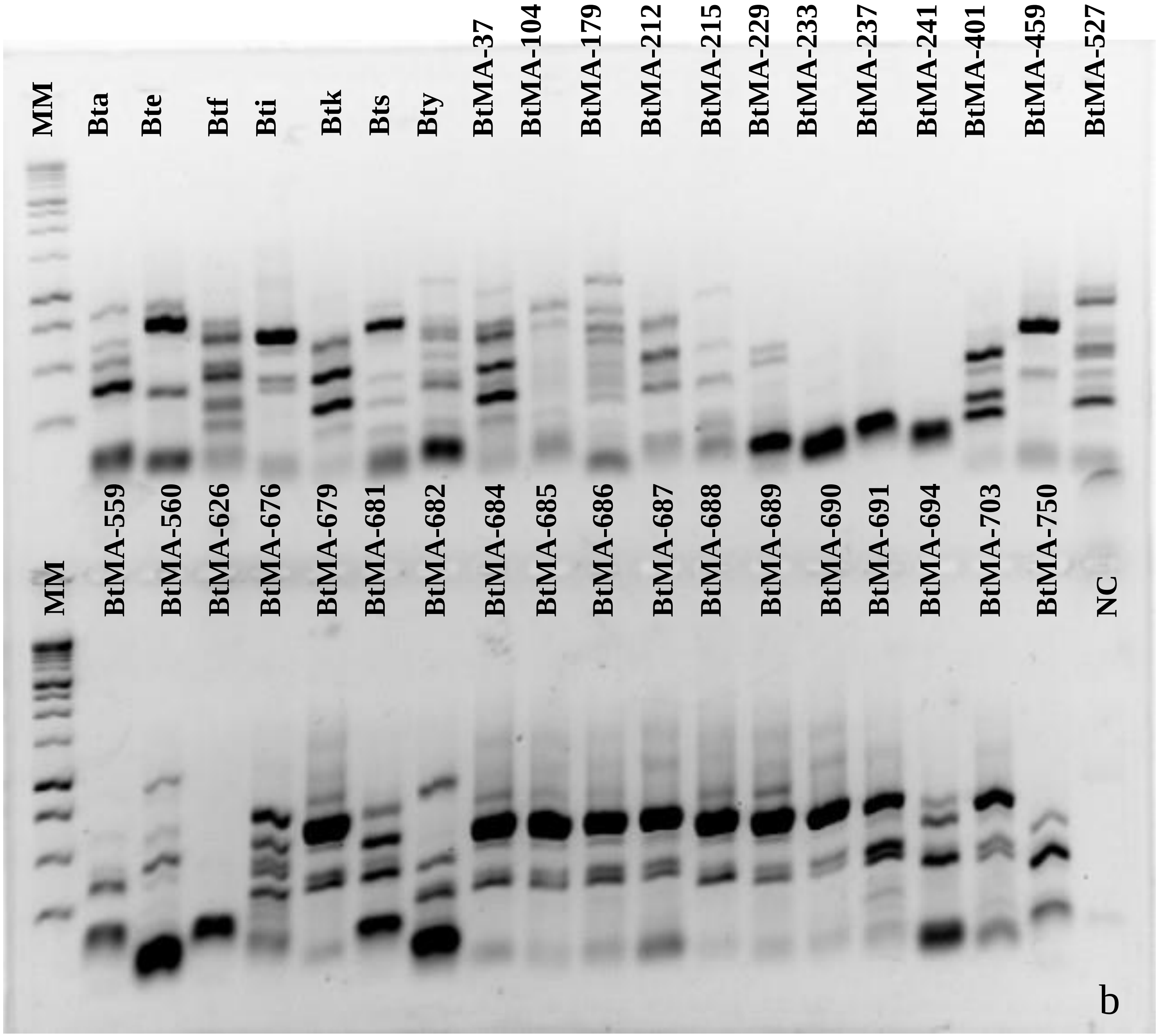
MM
Bta
Bte
Btf
Bti
Btk
Bts
Bty
BtMA-37
BtMA-104
BtMA-179
BtMA-212
BtMA-215
BtMA-229
BtMA-233
BtMA-237
BtMA-241
BtMA-401
BtMA-459
BtMA-527
MM
BtMA-559
BtMA-560
BtMA-626
BtMA-676
BtMA-679
BtMA-681
BtMA-682
BtMA-684
BtMA-685
BtMA-686
BtMA-687
BtMA-688
BtMA-689
BtMA-690
BtMA-691
BtMA-694
BtMA-703
BtMA-750
NC
b

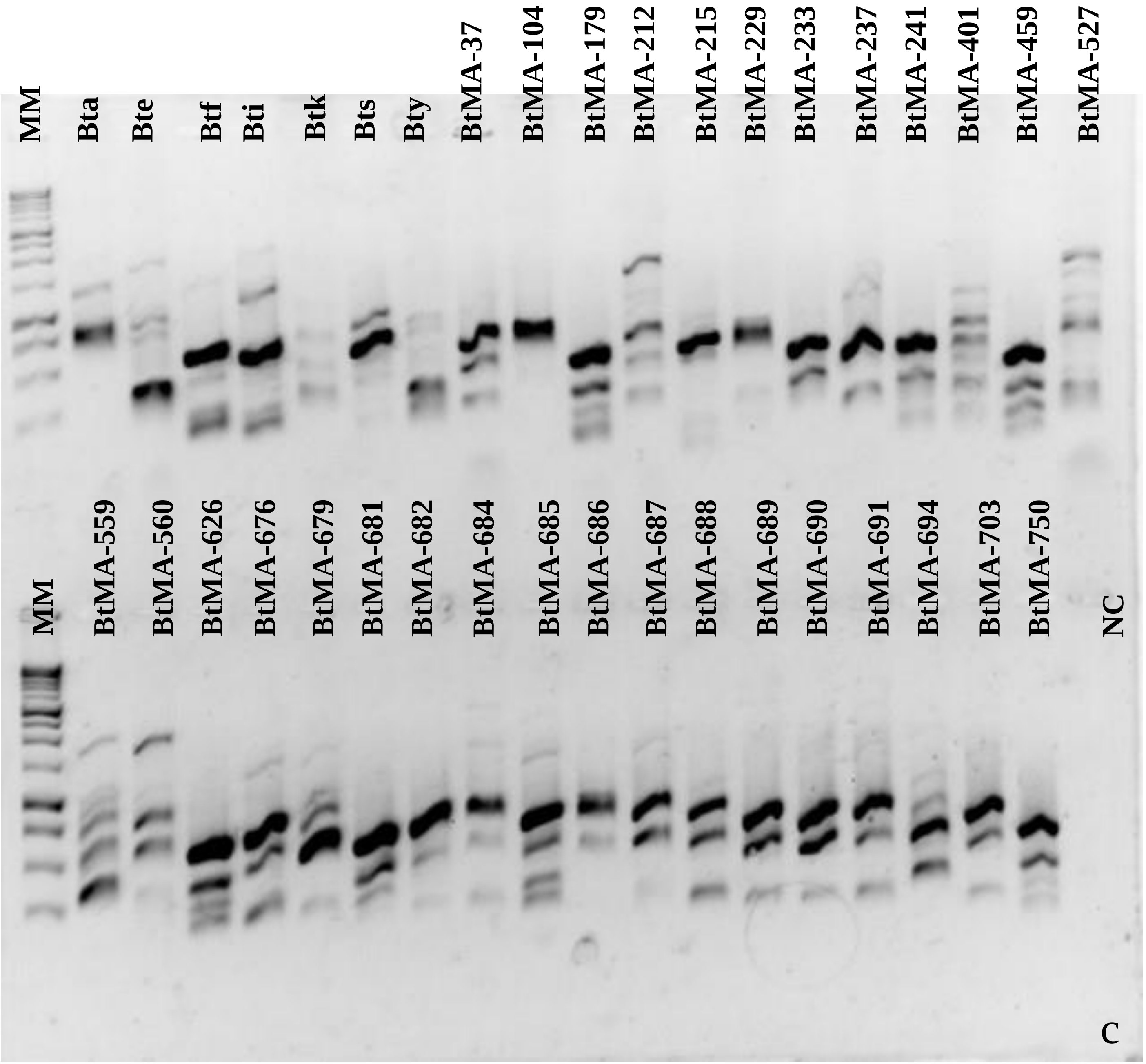
MM
Bta
Bte
Btf
Bti
Btk
Bts
Bty
BtMA-37
BtMA-104
BtMA-179
BtMA-212
BtMA-215
BtMA-229
BtMA-233
BtMA-237
BtMA-241
BtMA-401
BtMA-459
BtMA-527
MM
BtMA-559
BtMA-560
BtMA-626
BtMA-676
BtMA-679
BtMA-681
BtMA-682
BtMA-684
BtMA-685
BtMA-686
BtMA-687
BtMA-688
BtMA-689
BtMA-690
BtMA-691
BtMA-694
BtMA-703
BtMA-750
NC
C

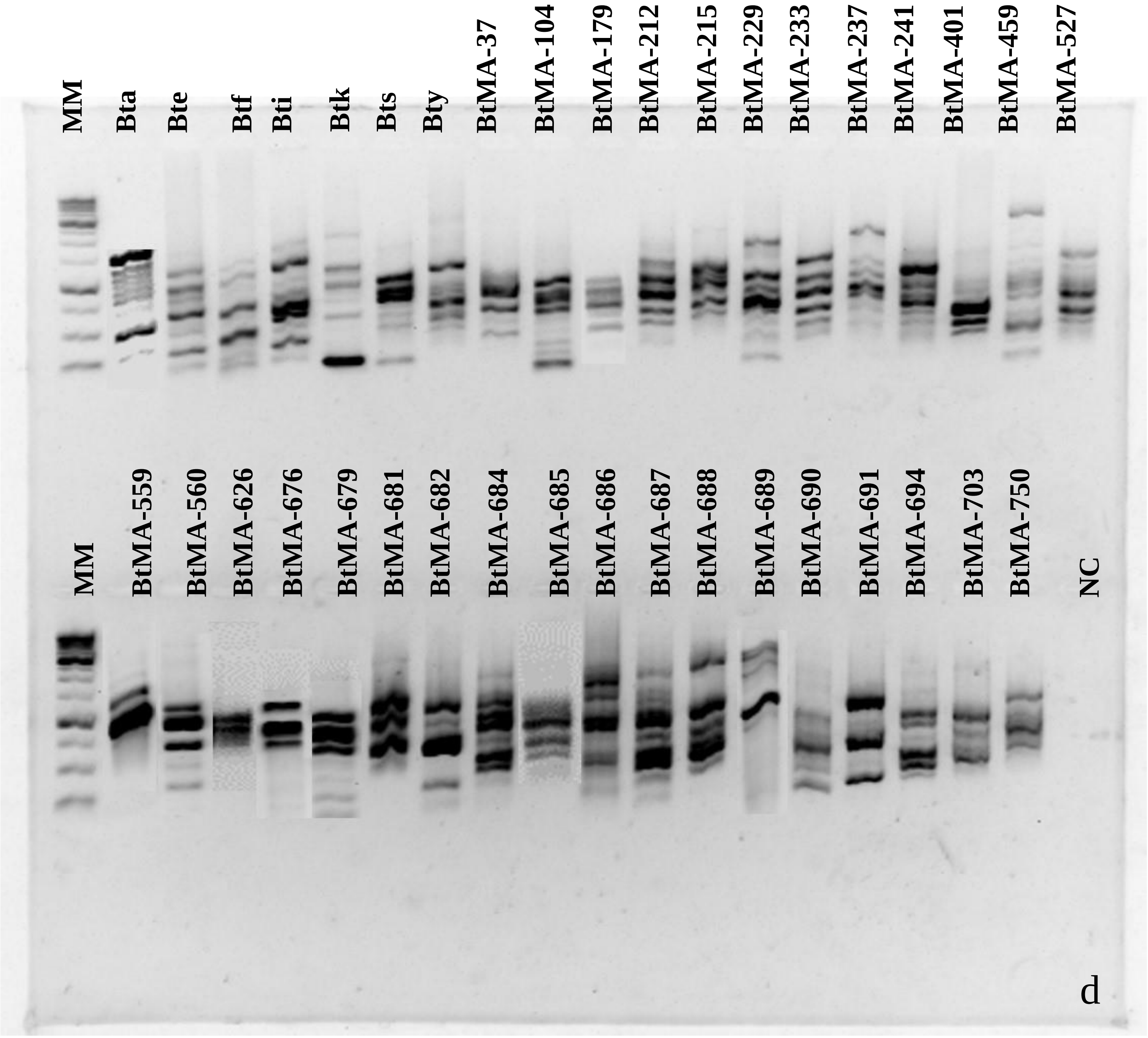
MM
Bta
Bte
Btf
Bti
Btk
Bts
Bty
BtMA-37
BtMA-104
BtMA-179
BtMA-212
BtMA-215
BtMA-229
BtMA-233
BtMA-237
BtMA-241
BtMA-401
BtMA-459
BtMA-527
MM
BtMA-559
BtMA-560
BtMA-626
BtMA-676
BtMA-679
BtMA-681
BtMA-682
BtMA-684
BtMA-685
BtMA-686
BtMA-687
BtMA-688
BtMA-689
BtMA-690
BtMA-691
BtMA-694
BtMA-703
BtMA-750
NC
d

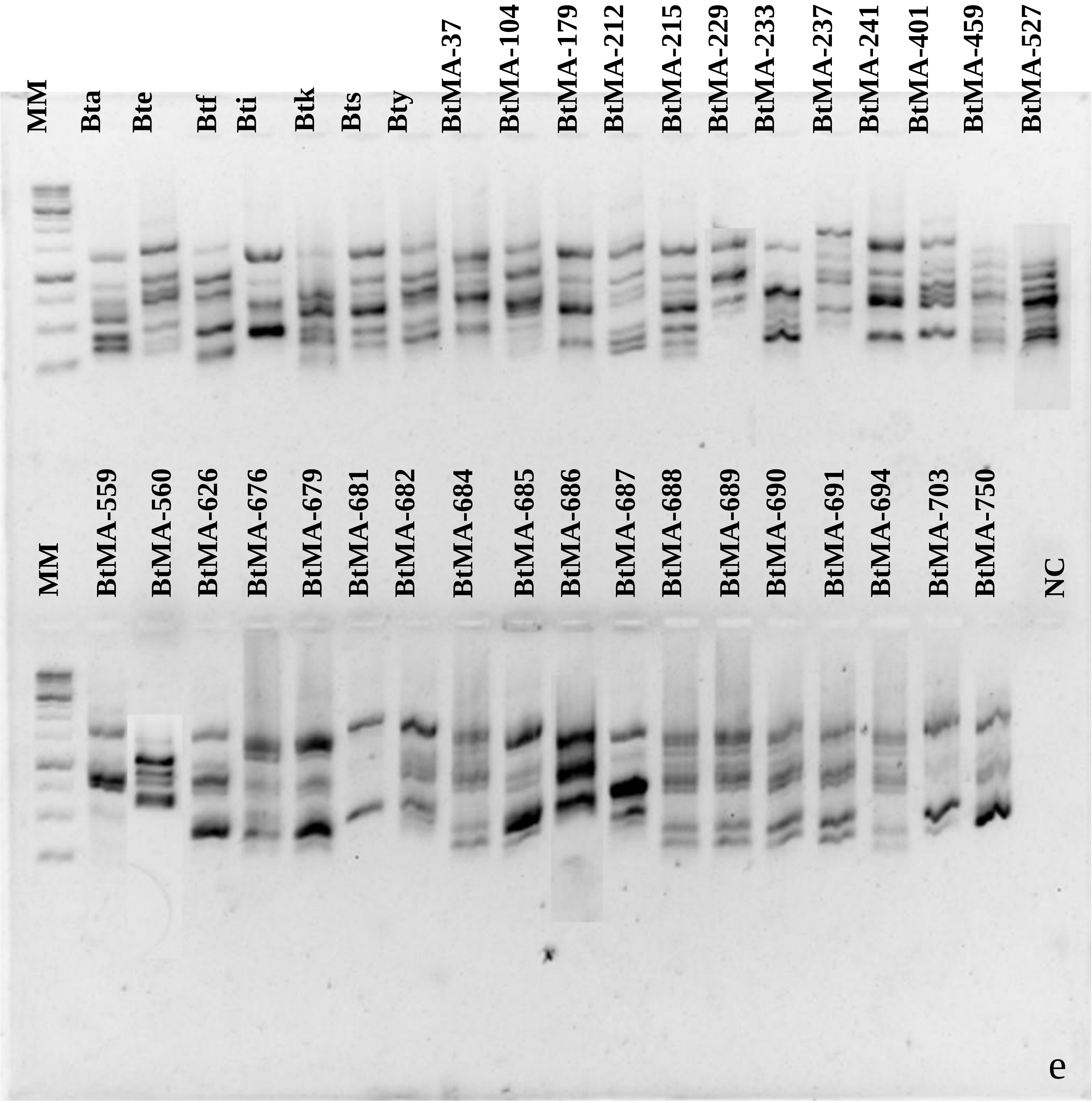
MM
Bta
Bte
Btf
Bti
Btk
Bts
Bty
BtMA-37
BtMA-104
BtMA-179
BtMA-212
BtMA-215
BtMA-229
BtMA-233
BtMA-237
BtMA-241
BtMA-401
BtMA-459
BtMA-527
MM
BtMA-559
BtMA-560
BtMA-626
BtMA-676
BtMA-679
BtMA-681
BtMA-682
BtMA-684
BtMA-685
BtMA-686
BtMA-687
BtMA-688
BtMA-689
BtMA-690
BtMA-691
BtMA-694
BtMA-703
BtMA-750
NC
e
